# Emergence of IFN-alpha TRAIL-expressing killer pDCs (IKpDCs) as a consequence of a crosstalk with NK cells. Influence of HIV-1 infection and implication of HMGB1

**DOI:** 10.1186/1742-4690-9-S1-P15

**Published:** 2012-05-25

**Authors:** Marlène Bras, Héla Saidi, Pauline Formaglio, Marie-Thérèse Melki, Marie-Lise Gougeon

**Affiliations:** 1 Institut Pasteur, Paris, France

## Background

Plasmacytoid dendritic cells (pDCs) mainly contribute to antiviral immunity through recognition of viral components resulting in the production of type-I interferon (IFN), a powerful innate antiviral cytokine. IFN-alpha production by pDCs is promoted by a cross-talk with NK cells, that triggers in return the cytotoxicity of NK cells. Given the essential role of pDCs and NK cells in viral control, we addressed the question of the impact of HIV on NK-pDC cross-talk, and the consequences on viral innate immunity.

## Methods

pDCs and NK cells were negatively sorted from PBMC of healthy donors. NK cells were kept either unstimulated (rNK) or activated with PMA/ionomycine for 2 hrs (aNK). pDC were either uninfected or infected with R5-HIV-1 BAL (pDCHIV) and cocultured with NK cells at different ratios for 24 h. The fate of both cell types was studied by multiparametric flow cytometry combined to Multianalyte Profiling technology.

## Results

HIV-1-infection of primary pDCs induced their maturation, characterized by the expression of maturation markers (HLA-DR, CD80, CD83, CD86) and the homing receptor CCR7. In addition, HIV-1 triggered the emergence of IFN-induced TRAIL expressing killer pDCs. The crosstalk of pDCHIV with aNK cells strongly increased the differentiation of pDCs into killer pDCs. Interestingly, the alarmin HMGB1, secreted by pDC upon HIV-1 infection, seems to be important in this cross-talk, since its modulation altered pDC maturation and the emergence of IKpDCs. At high concentrations of HIV-1, pDCs were able to activate rNK cells, as assessed by CD69 expression, and to induce IFN-gamma and TNF-alpha expression as well as perforin degranulation by aNK cells.

## Conclusion

We report for the first time that NK-pDCHIV crosstalk potentiates the emergence of TRAIL-expressing IFN-alpha-producing pDCs, and also triggers beta-chemokines synthesis and NK cell killing activity in a HMGB1 dependent manner. Overall these data suggest that the dialogue of HIV-infected pDCs with NK cells favors the emergence of both killer pDC and cytotoxic NK cells and promotes host innate immunity through the activation of potent antiviral effectors.

